# Comparisons among barley–pea mixed crop combinations in a replacement design as related to N fertilization and soil variation

**DOI:** 10.1038/s41598-023-43050-9

**Published:** 2023-09-22

**Authors:** Stefano Tavoletti, Stefania Cocco, Giuseppe Corti

**Affiliations:** 1 Dipartimento di Scienze Agrarie, Alimentari e Ambientali, Università Politecnica Delle Marche, Ancona, Italy; 2 Centro di Ricerca Agricoltura e Ambiente, Consiglio per la Ricerca in Agricoltura e l’analisi dell’Economia Agraria, Rome, Italy

**Keywords:** Plant breeding, Plant ecology, Ecology, Plant sciences

## Abstract

Two field trials (2017 and 2018) evaluated the performance of barley–pea mixed cropping by comparing different sowing densities (replacement design) and tailoring N fertilization on barley sowing density (split-plot design). High and Low N inputs were applied to whole plots whereas barley and pea, as pure and in mixed crops, were applied to subplots. The 2017 trial suggested the occurrence of an interaction between soil physical properties and N fertilization. Therefore, in 2018 a pedological survey allowed the soil effect to be included in the ANOVA model applied to evaluate crop performance parameters, showing that N fertilization positively affected barley performance only in the soil unit located downslope. A significantly lower presence of weeds was observed in mixed crops rather than in pea pure crops. Overall, increasing pea density and reducing barley density in mixed crops, and tailoring N fertilization were effective approaches to obtain a more balanced mixed grain at harvest. The combination of crop performance evaluation and assessments of soil conditions suggested that more sustainable agricultural systems, based on mixed cropping and a significant reduction of N fertilizers and herbicides, can be achieved with barley–pea mixed cropping as an alternative to pure cropping systems.

## Introduction

Sustainable intensification of production systems^1–5^ requires interdisciplinary approaches to manage the complexity of factors and interactions characterizing agricultural production processes^6–14^. The harmonization of cropping system and pedo-climatic conditions^15,16^, as well as the increase of biodiversity and the balancing among soil conditions, crop production, and animal farming could trigger the transition from conventional to sustainable farming systems^17–20^. For this purpose, the reintroduction of grain legume crops (for feed and/or food) deserves particular attention, although low market prices and low competitive ability against weeds restrain this achievement^21–24^, and cereal-legume intercropping shows beneficial effects for both environment and farmers because of their complementarity in N exploitation, reduction in the use of fertilizers and pesticides, and increased yield and quality traits of the final products^25–39^.

Intercropping grain legumes with cereals such as barley (*Hordeum vulgare* L.) or wheat [*Triticum turgidum* ssp. *durum* (Desf.) Husn. and *Triticum aestivum* L. subsp. *aestivum*] could appeal to farmers because of the agronomic advantages of intercropping compared with sole legume crops, especially in low-input and organic rainfed farming systems^26^. Intercropping grain legumes such as fababean (*Vicia faba* L.) or pea (*Pisum sativum* L.) with cereals (i) significantly reduces the presence of weeds compared with pure legume crops, thus avoiding/reducing herbicide treatments^34,40–42^, (ii) exploits interspecific complementarity^43,44^, (iii) takes advantage of intraspecific diversity^45^, (iv) improves land productivity and soil use efficiency as summarized by the Land Equivalent Ratio index^46,47^ and (v) reduces the infestations of parasitic plants such as *Orobanche* spp.^48,49^. Despite these advantages, intercropping has not spread on a large scale, prominently because of the need to separate cereal and legume grains after harvesting^26^. However, organic farmers exploit cereal-legume intercropping more than conventional farmers, due to the superior weed suppression than grain legume pure crops when herbicides are not used^34,50–53^.

Concerning N fertilization, mixed cropping could be a strategy to reduce N fertilization levels per unit area, as compared to cereal pure crops, if N supply would be tailored based on cereal crop density. Moreover, soil physicochemical properties along the soil profile and the asphyxial condition severity, characterizing silty-clay soils developed from thinly layered parent materials, can deeply affect crop performance^54,55^.

The present research deals with barley–pea intercropping, which is a very promising strategy for mixed farming systems in hilly soils submitted to a Mediterranean climate. Field trials were performed for two successive years (2017 and 2018) on a fine-textured and sub-alkaline soil characterized by a mild slope under a Mediterranean type of climate (central Italy). During the 2017 trial, we observed that soil location (upslope and downslope) could have affected crop performance as a response to N fertilization levels. Since the combination of soil parent material and topography could produce great soil spatial variability^56,57^ also at the experimental field level^58,59^, in 2018 a detailed investigation of soil physicochemical properties and profile was carried out to evaluate potential effects on crop performance.

Therefore, this research aimed at: (i) optimizing the overall performance of barley–pea mixed cropping by the evaluation of the relative performance of the two species at different sowing density ratios; (ii) evaluating the effectiveness of barley–pea mixed cropping to reduce N fertilizers by tailoring the N fertilization to barley sowing density in mixed cropping; (iii) considering soil profile variability to improve the evaluation of barley–pea performance in hilly soils.

## Materials and methods

### General information

Two field trials were carried out in 2017 and 2018 at the Experimental Farm of the Università Politecnica delle Marche (UNIVPM), Ancona, central Italy. The experimental area was in the lower portion of a hilly slope at an altitude between 46 and 48 m, with a S-SE exposure and gentle inclination (< 5%). Here, soils developed from semi-coherent and sub-alkaline Plio-pleistocene marine sediments, have a thickness that is usually below 1 m and a silty clay loam texture, and show rather frequent formation of shallow *vertic* cracks over the years. The experimental farm is equipped with a meteorological station that measures temperature and precipitation. Over the prior 30 years, the mean annual air temperature has been 13.3 °C, with January as the coldest month (4.5 °C) and July and August as the warmest ones (22.4 °C); the mean annual precipitation is 778 mm, which is mostly concentrated in autumn, with frequent summer droughts. A climatic diagram of the 30-year period and weather conditions in the years 2017 and 2018 are provided in Supplementary Figure S1.

### N fertilization strategy

Cereals and legumes differ for N fertilization requests because cereals use mineral N whereas legumes are characterized by N_2_-symbiotic fixation. For this reason, the choice of N fertilization level is an important aspect when contrasting pure and mixed crops, because intercropping could be a strategy to reduce N fertilization levels compared with pure crop-based conventional agriculture^60^. Therefore, for both field trials two levels of N (urea 46% N) fertilization were applied: High N and Low N. As shown in Supplementary Table S1, for crops involving barley the supplied amount of urea was tuned based on the barley sowing density, and the Low N level was set at 50% of the High N supplied to the corresponding crop (pure or mixed). For pea pure crop, 25 kg N ha^−1^ were supplied as High N whereas no N was applied at Low N level. In both field trials (2017 and 2018), a split-plot experimental design was established, including N fertilization as a whole plot factor (with two levels) and crops (pure and mixed crops) as subplot factor.

### First field trial: 2017

The 2017 trial (43° 32′ 40.96″ N–13° 21′ 33.64″ E) compared barley and pea pure crops with one mixed crop combination, named Mix1 (50:50). In the mixed crop, barley and pea were sown mixed in the same rows at 50% of their respective sowing densities as pure crops (350 and 80 plants m^−2^, respectively), therefore achieving plant densities of 175 (barley) and 40 (pea) plants m^−2^ in Mix1 (50:50). The 50:50 replacement combination has already been applied in several studies on barley–pea intercropping, with barley and pea sown mixed in the same row or sown in alternate rows at different arrangements^39,42,43,46,61^.

The trial was sown on February 17, 2017, as a split-plot in a randomized complete block design with five replications, a plot area of 10.8 m^2^ (9 × 1.2 m) with eight rows spaced 15 cm apart and N fertilization (NF) applied to the whole plots. Two barley (Tea and Sunshine, hereinafter coded as Barley1 and Barley2, respectively) and two pea (Hardy and Audit, hereinafter coded as Pea1 and Pea2, respectively) varieties were evaluated as pure crops and in Mix1 (50:50), mixed crops including all the four combinations between the two barley and the two pea varieties. On February 22, a pre-emergence herbicide treatment (Pendimetalin, STOMP AQUA 2.5 L ha^−1^) was applied, and N fertilization (urea 46% N) was supplied on April 11 using the experimental seeder with the drilling parts removed, so that the differential doses of N could be uniformly and individually supplied to each plot (Supplementary Table S1). The trial was harvested on July 21 using a combine harvester for small plots (Wintersteiger Delta). Certified commercial seed of all barley and pea varieties was used for both field trials performed in 2017 and 2018.

### Second field trial: 2018

In 2018, the field trial (43° 32′ 41.89″ N–13° 21′ 45.72″ E) was performed using a split-plot in randomized complete block design with four replications with the same plot size used in 2017. N fertilization (High N and Low N) was applied as whole plot factor and crops (pure and mixed crops) as subplot factor. Based on 2017 results, a wider range of plant teams was chosen in 2018 to test if a progressive reduction of barley density, together with a corresponding increase in pea density, could lead to a better performance of the mixed crop in terms of a more balanced harvested barley–pea grain mixture. Due to the higher number of mixed crop combinations, only the Tea barley variety (Barley1), the best performing one in 2017, was included in 2018. Moreover, the Astronaute pea variety (Pea3), being widely grown by local farmers, replaced Audit (Pea2), whereas Hardy (Pea1) was still included in the field trial. Overall, barley and pea were evaluated as pure crops and in four barley–pea mixed crop combinations: Mix1 (50:50), Mix2 (33:67), Mix3 (25:75), and Mix4 (20:80). Expressed as number of plants per m^2^, the expected plant densities of barley and pea in the mixed crops were: Mix1 (175:40), Mix2 (116:54), Mix3 (88:60), and Mix4 (70:64). The Mix1 (50:50), already tested in 2017, was retained as a control between the two years.

The trial was sown on February 1, 2018, and N fertilization was applied on April 17 of the same year. As in 2017, the amount of urea (46% N) applied to each mixed crop was reduced based on the relative density of barley in the four mixed cop combinations, as summarized in Supplementary Table S1, and pea pure crops received 25 and 0 kg of N per ha at the High and Low N level, respectively.

In 2018, to test the competitive ability of pure and mixed crops against weeds, no chemical herbicide treatment was applied, only the presence of *Convolvulus arvensis* L. and *Fallopia convolvulus* (L.) Á. Löve being controlled by manual weeding. The trial was harvested on July 3 using a combine harvester (Wintersteiger Delta).

### Traits evaluated in the field trials

In 2017 and 2018, pure and mixed crop grain yields (Mg ha^−1^) at 13% moisture were obtained. For the mixed crops, barley and pea grains were separated by sieving to obtain the grain yield of each species in the mixture. Moreover, the performance of mixed crops, as compared to pure crops, was evaluated by the Land Equivalent Ratio (LER) index^47,62^, which was calculated separately for barley (LER_b_) and pea (LER_p_), and as total LER (LER_tot_), as follows:

LER_b_ = LER for barley = Y_bmix_/Y_bpure_ = barley yield in mixed crop/barley yield in pure crop;

LER_p_ = LER for pea = Y_pmix_/Y_ppure_ = pea yield in mixed crop/pea yield in pure crop;

LER_tot_ = LER_b_ + LER_p_.

As reported by Mead and Willey^63^, the LER index is defined as “the relative land area required as pure crops to produce the same yields as intercropping”; therefore, when LER_tot_ > 1 it “represents the increased biological efficiency” of intercropping in specific environmental conditions.

In this research the observed LER values were also compared with expected ones based on the relative density of each species in the mixed crop combination. For example, if barley pure crop sowing density is 350 seeds m^−2^ and in a replacement design barley it is included at 50% of the sowing density of the pure crop (175 seeds m^−2^), if there is no difference in grain yield between barley grown in pure or in mixed cropping, the expected yield in mixed cropping would be equal to 50% the yield of barley grown as pure crop. Therefore, the relative densities of barley in the mixed crop combination (e.g. 50%) can be considered as the barley expected LER_b_ value (exp. LER_b_ = 0.50).

In 2017 and 2018, from pre-harvest sampling areas within each plot (1.2 m^2^), the number of plants m^−2^, crop dry matter yield (g m^−2^), number of spikes/pods per plant, and grain yield (g) per plant were determined for both barley and pea. These traits were considered to quantitatively evaluate the relative competitive ability of barley and pea in mixed cropping. Moreover, in 2018, due to the greater number of mixed crop combinations, also the Yield Ratio (barley yield/pea yield) was considered, after natural Log transformation, by Analysis of Variance (ANOVA) followed by multiple comparisons among means (Tukey’s HSD test, α = 0.05). For Yield Ratio, means will be reported after the inverse exponential transformation.

For traits collected from pre-harvest sampling areas, the ratio between values shown in mixed cropping and in pure crop was named as Equivalent Ratio (ER). The ER index represents the relative performance in mixed cropping for traits related with the competitive ability of the cereal and the legume in the different mixed crop combinations. Since a replacement design was applied both in 2017 and 2018, for number of plants per m^2^ and for crop dry matter yield (g m^−2^), the relative sowing density of barley and pea in each Mix was considered as the expected ER value. Differently, for number of spikes/pods per plant and for grain yield (g) per plant, the expected ER = 1 was applied.

### Soil property assessment in 2018

Based on 2017 results, in 2018 an assessment of soil properties was carried out together with the evaluation of pure and mixed crops performance. The soil of the 2018 field trial showed a slight inclination (2–4%) and the four blocks were placed following the field slope, from the highest altitude of Block 1 (approximately 48 m) to the lowest of Block 4 (approximately 46 m). Before sowing, on January 26, five soil trenches were dug in correspondence with the corridors (1 m wide) that then would have separated the blocks along the slope, so that each block was embraced by two profiles. Each soil profile was morphologically described following Schoeneberger et al.^64^ and sampled in large amounts by genetic horizons (approximately 1.5 kg per horizon). At a few meters from each trench, still in the corridor, 1–2 manual auger holes were drilled to replicate the observations for horizon thickness and depths at which slightly asphyxial conditions (OXI) and severely asphyxial conditions (UCTI) appeared.

Once in the laboratory, the soil samples were air-dried and passed through a 2-mm sieve to remove soil skeletal particles prior to being submitted to analyses. The pH was determined potentiometrically in water using a combined glass-calomel electrode at a solid:liquid ratio of 1:2.5 with a Crison pH-meter. The particle-size distribution was determined by the pipette method^65^ after the soil was disaggregated in distilled water for 24 h; the sand fraction (2–0.05 mm) was recovered by wet sieving, whereas silt (0.05–0.002 mm) was separated from clay (< 0.002 mm) by sedimentation while the columns were maintained at 19–20 °C. The total NH_4_–N content was determined by the semi-micro Kjeldahl method, while the soil organic carbon (SOC) content was estimated by the Walkley–Black method without the application of heat^66^. Potentially plant-available P was determined per Olsen et al.^67^.

### Field trial 2017: statistical analysis

Barley and pea were separately analyzed by the following fixed ANOVA model:

$$ {\text{y}}_{{{\text{ijk}}}} = {\text{m}} + {\text{a}}_{{\text{i}}} + {\text{b}}_{{\text{j}}} + {\text{e}}_{{{\text{ij}}}} + {\text{g}}_{{\text{k}}} + {\text{ag}}_{{{\text{ik}}}} + {\text{e}}_{{{\text{ijk}}}} $$

where y_ijk_ = traits evaluated (grain yield and LER, traits evaluated on pre-harvest sampling areas and ER); μ = overall mean; α_i_ = Nitrogen Fertilization (NF) effect (i = 1, 2); β_j_ = Block (B) effect (j = 1, …, 5); ε_ij_ = error 1 (NF × B interaction); γ_k_ = Plant Team (PT) effect, which for measured traits k = 1, …, 6 (2 pure crops + 4 mixed crops), whereas for LER values k = 1, …, 4 (4 mixed crops); αγ_ik_ = NF × PT interaction; ε_ijk_ = residual error.

Comparisons among means were performed using t test (α = 0.05) or multiple comparisons (Tukey’s HSD, α = 0.05). Pairwise contrasts (Bonferroni correction) between means were evaluated if requested for specific comparisons involving interactions.

The difference of LER_b_, LER_p_, and LER_tot_ from their respective expected values was tested by confidence interval calculated for observed values applying the studentized range coefficient q_α,k,ν_ (α = 0.05, k = number of means, ν = degrees of freedom of residual error mean square). If the confidence interval did not include the expected LER value, the observed LER value was statistically different from the expected one. The same approach was followed to compare ER observed values to ER expected ones. Data analysis^68–70^ was carried out using the software JMP 11.0.0. (SAS Institute).

### Field trial 2018: soil data analysis

Principal component analysis (PCA) and Cluster analysis (CA) were applied (NTSYS 2.02i software^71^) to analyze soil standardized data^70–72^. Each profile was characterized by physical variables (horizon thickness and texture, together with OXI and UCTI asphyxia depth) and soil chemical parameters measured on each horizon (pH, SOC, total NH_4_–N content, available P). Multivariate analyses (PCA and CA) were separately applied for physical and chemical variables. Concerning the soil fractions, only the percentages of silt and clay were included in the PCA and CA because the sum of the three particle-size fractions is always equal to 100%. To perform PCA and CA, the five soil profiles were considered as classification variables and Operational Taxonomic Units (OTUs), respectively.

For PCA, eigenvalues and eigenvectors of the Pearson’s correlation matrix were obtained, and a subset of the most important principal components (PCs) was retained based on the percentage of total variance explained. For the chosen PCs, the scores of each soil profile were plotted to graphically evaluate whether some soil profile trend characterized the slope of the experimental field.

The squared loadings of the variables, being measured for all profiles, allowed the evaluation of the relative importance of each horizon for the differentiation among soil profiles detected by PCA. In PCA, the eigenvalues are the variances explained by each PC and, for each PC, the sum of squared loadings of all measured variables equals the PC eigenvalue. Therefore, the sum of squared loadings of variables measured for each horizon is the contribution of each horizon to the total PC variance. Consequently, the ratio between the sum of the squared loadings divided by the PC eigenvalue provided the relative contribution (% of eigenvalue) of each horizon to the total variance of each PC. This ratio was applied to quantify the relative importance of each horizon and of asphyxia depth for the differentiation among profiles within each selected PC.

Furthermore, cluster analysis (CA), based on the Euclidean matrix of distances between profiles and the Unweighted Pair Group Method with Arithmetic mean (UPGMA) clustering method, was applied to complement the PCA results.

### Field trial 2018: crop data analysis

Due to the higher number of mixed crop combinations evaluated in 2018 than in 2017, the data analysis of all traits but dry matter of weeds was separately performed for pure and mixed crops by applying two different ANOVA models (Supplementary text file S1). The PCA and CA results, concerning soil homogeneity along the slope, highlighted a clear differentiation between the upslope (including Blocks 1 and 2) and the downslope (including Blocks 3 and 4) areas of the experimental field. Therefore, the Soil factor was added into ANOVA models with two levels: Soil1 (upslope) and Soil2 (downslope). Consequently, Blocks 1 and 2 were nested within Soil1 whereas Blocks 3 and 4 were nested within Soil 2. Having added the Soil factor was equivalent to split the field trials in two areas (Soil1 and Soil2), each including two complete Blocks. Therefore, ANOVA is equivalent to the model usually applied for the analysis of factorial experiments carried out in different locations, Soil1 and Soil2 in the present trial, in a split-plot arrangement in each location (NF as whole plot factor and crops, pure and mixed crops as subplot factor).

For the pure crops (Plant Team factor with 3 levels), the analysis of grain yield and traits collected on sampling areas was performed applying “ANOVA Model A” shown in the Supplementary text file S1. The same Model A was applied to analyze the dry matter of weeds including both pure and mixed crops (Plant Team factor with 11 levels, 3 pure, and 8 mixed crops). The F tests were performed using the following mean squares as denominator: (i) “Blocks nested within Soil” mean square to test the Soil mean square, (ii) “Blocks × NF nested within Soil” to test the NF and NF × Soil mean squares, and (iii) the residual error to test Plant Team (PT) and Mix main effects and their first and second order interactions.

For mixed crops, the Mix factor with 4 levels (Mix1 to Mix4) was in a factorial arrangement with Plant Team (PT) factor with 2 levels (Barley1–Pea1 and Barley1–Pea3). Therefore, the Mix (4 levels) and PT (2 levels) factors, and their interaction, were included in ANOVA model. Overall, for mixed crops a fully factorial arrangement of Soil, Nitrogen Fertilization (NF), Plant Team (PT), and Mix fixed factors was applied, with Blocks nested within Soil.

To analyze each species in mixed cropping for the grain yield and traits collected on sampling areas, the “ANOVA Model B” was applied (Supplementary text file S1). The F tests were performed using as denominator mean square: (i) “Blocks nested within Soil” mean square to test the Soil mean square, (ii) “Blocks × NF nested within Soil” to test the NF and NF × Soil mean squares, and (iii) the residual error to test PT and MIX main effects, and their first, second, and third order interactions. The overall performance of mixed crops was evaluated by the analysis of total yield, LER_tot_, and Yield Ratio. Multiple comparisons among means and pairwise contrasts were performed as described for the 2017 field trial.

The “ANOVA model B” was also applied to obtain the respective standard error of the LER_b_, LER_p_, LER_tot_ and ER means, for each trait analyzed. This allowed to test the statistical difference of each LER and ER value from the respective expected value by using the confidence interval of observed means, obtained using the studentized range coefficient q_α,k,ν_ (α, k = number of means, ν = degrees of freedom of residual error mean square).

### Legal statement

The present study complies with relevant institutional, national, and international guidelines and legislation. The research performed field studies using commercially available plant varieties, therefore officially certified, of barley (2 varieties) and pea (3 varieties), all varieties being registered in the European Common Catalogue of varieties of agricultural plant species. No appropriate permissions and/or licences for collection of plant or seed specimens were required to conduct this research.

## Results

### Field trial 2017

The ANOVA for grain yield (Supplementary Table S2) showed a highly significant Plant Team (PT) mean square for both barley and pea, whereas NF and PT × NF interactions were not significant. All pure crops had a very good performance in 2017, based on average performance of barley and pea in the area where the field trial was performed (Table 1). In mixed cropping, barley yield was always significantly lower than pure Barley1 and lower than pure Barley2 only in mix combination with Pea1. However, all LER_b_ values (range 0.71–0.88) were significantly higher than expected (expected LER_b_ = 0.50), reflecting a very good performance of the cereal in mixed cropping, given that the sowing density of barley was half that of the pure crop.

**Table 1:** Field trial 2017: multiple comparisons (Tukey’s HSD test, α = 0.05) for grain yield of barley and pea in mixed cropping and comparison between observed and expected LER values (LER_b_, LER_p_, and LER_tot_) performed by the use of the confidence interval of observed values (studentized range coefficient).

Plant Team	Barley^1^	LER_b_^2^	Exp. LER_b_	Pea^1^	LER_p_^2^	Exp. LER_p_	LER_tot_^2^	Exp LER_tot_
Pea1 (Hardy)				3.88a				
Pea2 (Audit)				3.06b				
Barley1 (Tea)	7.54a							
Barley2 (Sunshine)	6.60b							
Barley1–Pea1	5.33c	0.71***	0.50	0.46c	0.12***	0.50	0.83***	1,00
Barley1–Pea2	5.78bc	0.77***	0.50	0.44c	0.11***	0.50	0.88**	1,00
Barley2–Pea1	5.69c	0.87***	0.50	0.46c	0.16***	0.50	1.03 ns	1,00
Barley2–Pea2	5.79bc	0.88***	0.50	0.43c	0.15***	0.50	1.03 ns	1,00

^1^Means followed by different letters are statistically different (Tukey’s HSD test, *P* < 0.05).

^2^The difference between observed and expected (Exp.) LER values of barley and pea in each mix was tested by the confidence interval of each observed LER applying the studentized range coefficient: ns, not significant; ***P < 0.001.

Pea yield in mixed cropping was always much lower than both pea pure crops, and LER_p_ values (range 0.12–0.14) were always significantly lower than expected (expected LER_p_ = 0.50), suggesting a very low performance of pea in mixed cropping with barley. Overall, in all mixed crop combinations barley performed much better than pea. Consequently, the LER_tot_ values of all mixed crops were relatively low (range 0.83–1.03) and significantly lower than 1 for the 2 mixed crops including Barley1, the highest yielding variety as pure crop.

Results on traits collected on sampling area are reported in Supplementary Table S3. The number of plants per m^2^ of mixed crops was significantly lower than the respective pure crops for both barley and pea. However, the plant densities of barley and pea were mostly close to their respective expected value (in Mix1, expected ER_b_ = expected ER_p_ = 0.50), only Barley2 and Pea2 showing one mixed crop combination with significantly higher and lower plant densities than expected, respectively.

Barley dry matter yield of most mixed crops was not significantly lower than that of barley pure crops, with all ER_b_ values significantly higher than expected (range 0.78–0.90), whereas pea dry matter yield was always significantly lower than pure crops resulting in very low and significantly lower than expected ER_p_ values (range 0.13–0.22).

Furthermore, the number of barley spikes per plant and grain yield per plant were significantly higher in mixed cropping than in pure crops, as confirmed by significant ER_b_ values (expected ER_b_ = 1). Therefore, lowering the plant density of barley in mixed cropping increased its tillering ability and consequently its dry matter yield was higher than expected. Differently, for pea the number of pods per plant and grain yield per plant were always significantly lower in mixed cropping than in the pure crops, as confirmed by significantly lower ER_p_ values than expected.

Overall, the 2017 results suggested that the Mix1(50:50) was not an efficient combination between the two species in terms of the respective performance and relative competitive ability of barley and pea in mixed cropping.

Concerning the ANOVA results, Blocks and NF main factors, as well as PT × NF interaction, were not significant for grain yield of both barley and pea. This result was expected for a legume, not for a cereal. Since the five blocks were located along the field slope, and for barley yield Blocks × NF mean square was highly significant (Supplementary Table S2), barley response to N fertilization could have been related to soil variability along the field slope. In central Italy, especially in hilly areas, soil spatial variability can be high but information about its influence on crop performance is scarce. Therefore, the 2018 field trial included the evaluation of soil asphyxial conditions and physicochemical properties together with a wider number of barley–pea mixed crop combinations.

### Field trial 2018: soil properties

The five soil profiles (Supplementary Table S4) displayed a sequence of Ap horizons (those interested by plowing) reaching a depth between 34 and 48 cm, followed by a Bw horizon with variable thickness, and a BCg horizon that extended to the depth of approximately 1 m. In-between the Ap horizons there was an Ap&Oi horizon formed by the last plowing, which occurred after the 2017 wheat harvesting. The undecomposed organic material (Oi) forming this horizon was perfectly recognizable as wheat straw, which was mixed with the mineral material upon resting at the soil surface. The presence of an Ap3 horizon underneath the Ap&Oi indicated that deeper plowings occurred in the past.

**Figure 1:**
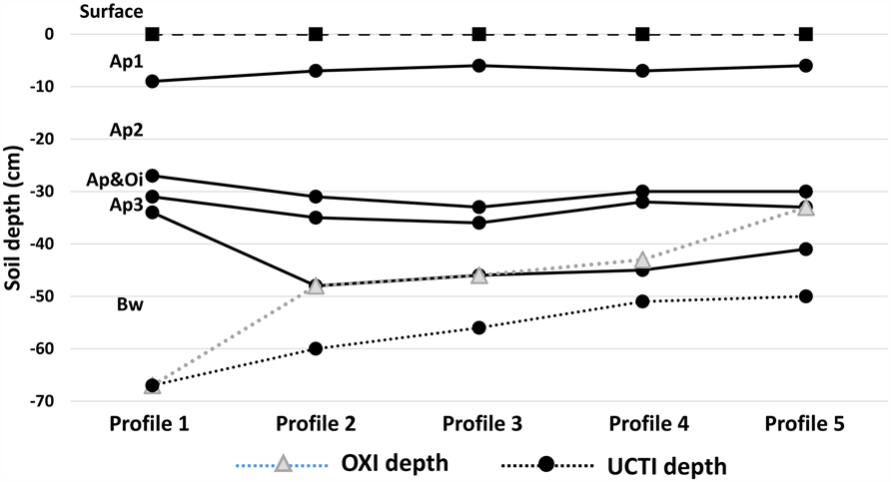
Horizon thickness of all profiles, including the depths at which the asphyxial horizon (OXI) and severely asphyxial horizon (UCTI) begin to appear.

Detailed observations of the profiles allowed us recognizing features revealing the existence of asphyxial conditions (Supplementary Table S4 and Fig. 1). These conditions occur to various extents in the soil and depend on the level of soil wetness. Only when certain thresholds of mottling and coloring are reached because of permanent or semi-permanent water saturation does the horizon experience more or less severe asphyxial conditions, which are indicated by the suffix “g” to the designation of the master horizon, which denotes *gley* conditions^73,74^. When these thresholds are not reached, the soil is subjected to mild asphyxial conditions, and the “g” attribution is not used. Mild asphyxial conditions were observed in our soil profiles, where mottles were present above the BCg horizon. Hence, we considered the horizons with signs of mottling as slightly asphyxial (OXI) and the BCg horizons where mottling was acute as severely asphyxial (UCTI). In the experimental field, we observed a progressive approach of the asphyxial conditions toward the surface going downslope, as expected by the progressive decline of altimetric gradient and inclination from profile 1 to profile 5 (Supplementary Table S4).

Soils were always sub-alkaline (mean pH = 8.1; range 7.8–8.2) and displayed a silty clay to silty clay loam texture, with only the Ap3 horizon of profile 4 having a clay texture. The contents of total NH_4_–N and SOC were very low (mean NH_4_–N = 1.07 g kg^−1^, range 0.80–1.35 g kg^−1^; mean SOC = 10.05 g kg^−1^, range 6.80–14.90 g kg^−1^). The available P content was also generally low, with only one horizon (Ap&O1 of profile 1) approaching 20 mg kg^−1^ (mean available *P* = 10.1 mg kg^−1^, range 1.0–20.6 mg kg^−1^). These results reflected the typical variability of hilly soils of central Italy^75–77^.

### Field trial 2018: soil data analysis

**Figure 2:**
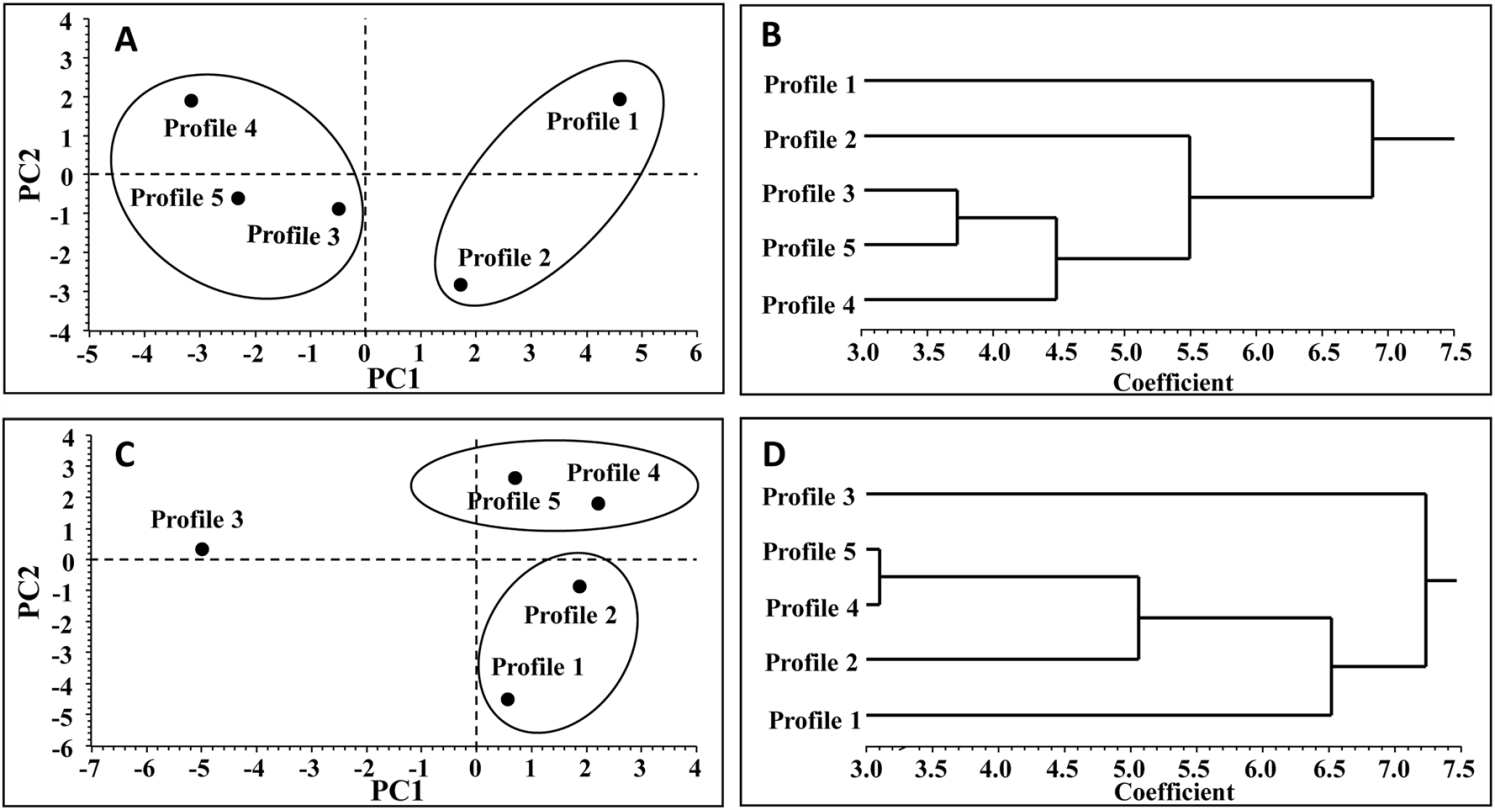
Multivariate analysis of soil profile variability. Scatterplot of principal component analysis (PCA) and dendrogram of cluster analysis (CA) for physical (**A**,**B**) and chemical (**C**,**D**) variables, respectively.

For physical parameters, 82.6% of total variance was explained by PC1 (58.4%) and PC2 (24.2%). The PC1 differentiated profiles 1 and 2, located upslope, from profiles 4 and 5, located downslope (Fig. 2A), with profile 3 being similar to profiles 4 and 5. Based on the evaluation of the PC1 squared loadings of each profile, most variation between upslope and downslope profiles was related to Ap&Oi and Bw horizons, together with the OXI and UCTI depths, whereas Ap1, Ap2, and Ap3 horizons explained most of the variation in PC2 scores among profiles (Supplementary Table S5). The dendrogram of cluster analysis (CA) confirmed, for physical variables, the differentiation between upslope (profiles 1 and 2) and downslope (profiles 4 and 5), profile 3 being intermediate but closer to profiles 4 and 5 than to profiles 1 and 2 (Fig. 2B).

Concerning chemical parameters, 81.8% of total variance was explained by PC1 (42.7%) and PC2 (39.1%). PC1 clearly differentiated profile 3 (negative score) from all other profiles (Fig. 2C), squared loadings showing that this difference was mainly due to the Ap1 and Ap3 horizons (Supplementary Table S5). PC2 separated profiles 1 and 2 (negative scores) from profiles 4 and 5 (positive scores), squared loadings highlighting that these differences were mainly related to Ap2, Ap&Oi, and Bw horizons. The CA dendrogram (Fig. 2D) for chemical parameters not only highlighted that profile 3 was clearly separated from all other profiles but confirmed, as also shown by physical parameters, that profiles 4 and 5 were more similar between each other than to the profiles 1 and 2.

Overall results of PCA and CA on physical and chemical variables highlighted a clear differentiation along the field slope between upslope and downslope soil profiles. This result allowed to subdivide the experimental field into two main units (named Soils): (i) Soil1, located upslope and including Profiles 1 and 2, and (ii) Soil2, located downslope and including Profiles 4 and 5. Profile 3 was considered as a landmark located between Soil1 and Soil2, setting a cutting line between the upslope and downslope parts of the field.

Supplementary text file S2 explains how the results on barley grain yield, obtained applying ANOVA model not including the Soil factor, reflected the independent results obtained from soil analysis. This comparison allowed the Soil factor to be subsequently included in the ANOVA models for a more comprehensive evaluation of pure and mixed crop performance in 2018.

### Field trial 2018: pure crop performance

PT main factor was highly significant for all 5 traits (Supplementary Table S6). However, since varieties of 2 very different species were included in the ANOVA for pure crops, comparisons between species concerning the number of spikes (barley) or pods (pea) per plant and for grain yield per plant were not further considered. For grain yield, significant first and second order interactions of PT with Soil and NF were detected, and a significant PT × NF interaction was found also for dry matter yield per m^2^.

In 2018, heavy rains and freezing temperatures occurring after sowing (Supplementary Figure S1) had negative effects on barley average grain yield, which was much lower than in 2017. As shown in Supplementary Table S7, average Barley1 grain yield was significantly lower than both Pea1 and Pea3, but PT × Soil × NF interaction showed that a significant increase of barley yield at High N was observed only in Soil2, not in Soil1. As pure crops, Pea3 had a significantly higher grain yield than Pea1 (PT main effect), but grain yield of both pea varieties was not affected by Soil and N fertilization. No significant difference due to Soil and/or NF were detected for all other traits but dry matter yield, that significantly increased only in Soil2 for Pea3 and Barley1. Therefore, variability between Soil1 (upslope) and Soil2 (downslope), highlighted by soil analysis, differently affected the response of barley and pea pure crops to N fertilization.

### Field trial 2018: mixed crops performance

The ANOVA results for mixed crops are reported in Supplementary Table S8. Much attention was addressed to evaluate the Mix main effect and its interactions with Soil and NF. Mix main factor was highly significant for all variables except total yield and number of pods per plant of pea. Mix × Soil × NF was significant only for barley grain yield, suggesting different trends of barley response among the 4 mix crop combinations in different Soils and at different NF levels. Moreover, the three-way interaction was also significant for barley grain yield, suggesting differences between the two barley–pea combinations across Mix, Soil and NF levels.

Multiple comparisons among Mix means showed that barley yield significantly decreased whereas pea yield significantly increased from Mix1 to Mix4 (Table 2). However, LER_b_ and LER_p_ values were significantly (*P* < 0.001) higher and lower, respectively, than expected LER values, reflecting a better performance of barley than pea in mixed cropping. At 50% sowing density (Mix1), barley retained 84% of pure crop average yield; at the lowest sowing densities of 25% (Mix3) and 20% (Mix4), barley still retained 59% (Mix3) and 54% (Mix4) of its yield as pure crop. An opposite trend was observed for pea, whose average grain yield increased from Mix1 to Mix4, but it was much lower than expected based on pea sowing density: from 29% (observed) *vs*. 50% (expected) in Mix1 to 51% (observed) *vs*. 80% (expected) in Mix4.

**Table 2:** Field trial 2018: mixed crops.

Mix	Barley			Pea		
	Yield^1^	LER_b_^2^	Exp. LER_b_	Yield^1^	LER_p_^2^	Exp. LER_p_
Mix1	2.17a	0.84***	0.50	0.87c	0.29***	0.50
Mix2	1.78b	0.70***	0.33	1.18b	0.40***	0.67
Mix3	1.50c	0.59***	0.25	1.49a	0.50***	0.75
Mix4	1.38d	0.54***	0.20	1.52a	0.51***	0.80

Mix main factor: multiple comparisons among mean grain yields (Mg ha^−1^) and LER values of barley and pea in the four mixed crop combinations (Mix1–Mix4).

^1^Means followed by different letters are statistically different (Tukey’s HSD test, *P* < 0.05).

^2^The difference between observed and expected (Exp.) LER values of barley and pea in each mix was tested by the confidence interval of each observed LER applying the studentized range coefficient: ***P < 0.001.

**Figure 3:**
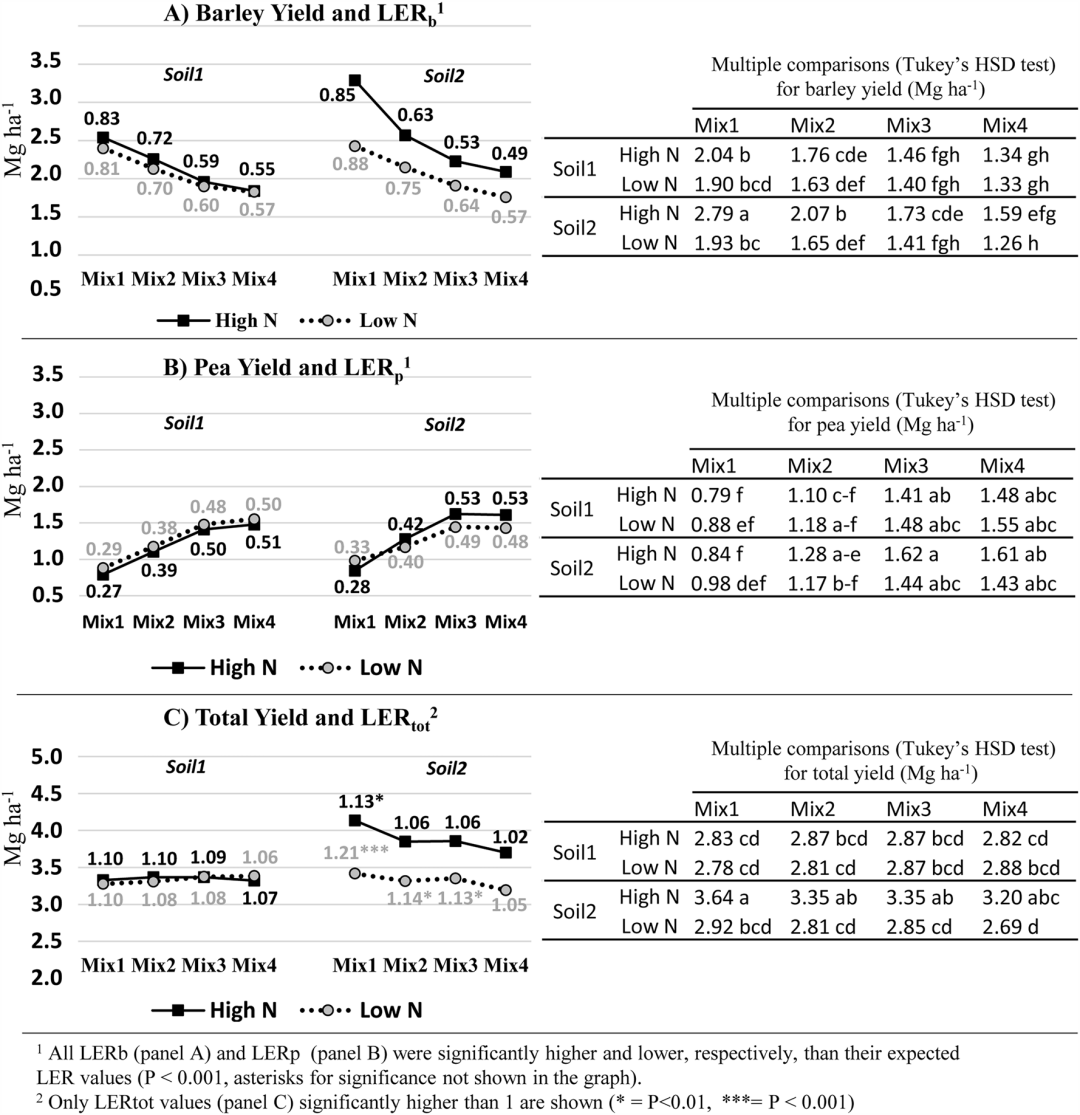
Field trial 2018. Results of Mix × Soil × NF interaction for grain yield: multiple comparisons among grain yield means and test (by confidence interval) of LER differences from expected values for (**A**) barley, (**B**) pea, (**C**) total yield.

The evaluation of Mix main factor supplied a general view of barley and pea average response to the differences in sowing densities characterizing the four mixed crop combinations. However, a more comprehensive picture of barley and pea grain yield performance in mixed cropping was provided by the significant Mix × Soil × NF interaction (Fig. 3).

As shown in Fig. 3A, in both soils barley yield significantly decreased from Mix1 to Mix4, but for each mixed crop combination significant differences between High N and Low N were detected in Soil2 (downslope), not in Soil1 (upslope). Moreover, all LER_b_ values were highly significant (*P* < 0.001), confirming that the barley performance, in terms of grain yield, was much higher than expected.

Results of Mix × Soil × NF interaction for pea yield and total yield are reported in Fig. 3B,C, for a comprehensive parallel evaluation between barley and pea performance, together with the whole performance of the mixed crops. Pea grain yield significantly increased from Mix1 to Mix4 with a similar trend in both soils, but no significant difference due to N fertilization or Soil was detected at each Mix level. However, all LER_p_ values were significantly lower (*P* < 0.001) than expected based on pea sowing density in each mixed crop combination, confirming that, in terms of grain yield, pea had a lower performance than barley in mixed cropping.

As shown in Fig. 3C, only in Soil2 (downslope) the total yield of each Mix was significantly higher at High N than at Low N. Moreover, within each Soil × NF level, no significant difference in total yield was observed from Mix1 to Mix4, because barley and pea yields balanced each other resulting in similar final total grain yields regardless of their relative sowing densities. All LER_tot_ values were higher than 1, although significant LER_tot_ values were detected only in Soil2 involving Mix1 at High N and Mix1, Mix2 or Mix3 at Low N. This result could be related to the significant difference in grain yield of pure barley between High N and Low N levels (Supplementary Table S7), resulting in lower LER_b_ values at High N than at Low N.

A significant three-way interaction (Mix × PT × Soil × NF) was obtained only for barley yield and evidenced only a slightly different response of barley to N fertilization between Barley1–Pea1 and Barley1–Pea3 in Soil2 (Supplementary Figure S2).

Overall, adding the Soil factor in ANOVA model pointed out that the performance of barley in different mixed crop combinations and the effectiveness of N fertilization on barley yield differed along the slope of the experimental field. Moreover, tuning N fertilization to barley sowing density showed that very good barley performance can be realized by lowering both the barley sowing densities and the supply of N fertilizers, as pointed out by the trend of LER_b_ values.

For an overall analysis of mixed crop effectiveness, the Yield Ratio (Supplementary Table S9) showed that pea and barley grains were equally represented in the final harvest of Mix3 and Mix4 (yield ratio close to 1), whereas in Mix1 and Mix2 barley grain was prevalent (yield ratio higher than 1). Significant differences in the Yield Ratio between Mix1 versus Mix3 and Mix4 were obtained. Therefore, Mix3 and Mix4 combinations could be considered as being characterized by an overall better performance than Mix1 and Mix2 in terms of getting a better balance between barley and pea grain at harvest, together with a slightly but not significantly lower total yield.

### Field trial 2018: competitive ability in mixed cropping

For both barley and pea, the average number of plants per m^2^ significantly decreased and increased from Mix1 to Mix4, respectively, but observed values reflected the expected sowing densities for all mixed crop, as confirmed by not significant ER_b_ and ER_p_ values (Supplementary Table S10).

The dry matter (g m^−2^) of barley and pea were respectively higher and lower than expected, as shown by the highly significant ER_b_ and ER_p_ values (Supplementary Table S10). Noteworthy is that the barley dry matter yield in Mix1 was very close to the value of the pure crop (ER_b_ = 0.88), and in Mix4 it was on average about 60% of the pure crop value (ER_b_ = 0.59), although the low barley sowing density (20%). Moreover, the average number of spikes per barley plant as well as the grain yield per plant were significantly higher in Mix3 and Mix4 than in Mix1 and the ER_b_ values were always significantly higher than 1 (Supplementary Table S10). Therefore, lowering the sowing density favored better tillering and grain yield of the barley plants, confirming that barley could reach very good performance in mixed cropping also at low sowing density. Differently, pea average number of pods per plant in all mixed crops did not significantly differ among mixed crop combinations, but it was always, as well as the grain yield per plant, significantly lower than the pure crop (expected ER_p_ = 1, Supplementary Table S10).

### Field trial 2018: dry matter of weeds

For weeds dry matter (g m^−2^), PT and PT × NF interaction mean squares were significant (Supplementary Table S6). Multiple comparisons (Table 3) showed that the presence of weeds in pea pure crops (131 and 121 g m^−2^ for Astronaute and Hardy, respectively) was significantly higher than in barley pure crop (60 g m^−2^) and in all mixed crop combinations (range 40–66 g m^−2^). Moreover, PT × NF interaction highlighted a significant increase of weeds due to High N fertilization only for pure pea crops (Table 3). These results confirmed the effectiveness of mixed cropping for the control of weeds as compared to pea pure crops. Moreover, supplying N fertilizer to pea pure crops did not increase grain yield but significantly increased the presence of weeds.

**Table 3:** Weeds dry matter (g m^−2^).

Plant Team	A	B		Contrast^2^
	PT main effect^1^	PT × NF interaction		
	Weeds (g m^−2^)	Low N	High N	
Pea3 (pure)	131.0a	99.7	162.2	*
Pea1 (pure)	120.7a	92.8	148.5	*
Mix1—(Pea1–Barley1)	66.0b	65.3	66.7	ns
Barley1 (pure)	60.4b	51.4	69.4	ns
Mix3—(Pea3–Barley1)	57.0b	48.5	65.6	ns
Mix4—(Pea1–Barley1)	55.8b	40.7	70.9	ns
Mix2—(Pea3–Barley1)	51.9b	54.5	49.3	ns
Mix3—(Pea1–Barley1)	47.9b	62.3	33.5	ns
Mix2—(Pea1–Barley1)	45.5b	50.7	40.2	ns
Mix4—(Pea3–Barley1)	43.7b	44.7	42.7	ns
Mix1—(Pea3–Barley1)	40.4b	38.0	42.7	ns

(A) multiple comparisons (Tukey’s HSD test) among means of pure and mixed crops, and (B) Contrast between weed means at High N and Low N level (PT × NF interaction).

^1^Means followed by different letters are statistically different (*P* < 0.05).

^2^For each plant team, the statistical significance of each contrast between High N and Low N levels is shown as: ns, not significant; *P < 0.05.

## Discussion

Small grain cereals in general are more competitive than grain legumes in mixed cropping^78–81^.

The field trial carried out in 2017 concerning Mix1 (50:50) confirmed that the level of tillering and the dry matter yield explained the highly competitive ability of barley in mixed cropping, although the plant density of barley reflected the expected sowing density. Differently, pea in Mix1 (50:50) was characterized by a much lower performance than expected based on sowing density. These results, as also previously reported^41,43,82^, suggested testing different mixed crop combinations other than Mix1.

The 2018 field trial showed that decreasing barley and increasing pea sowing density led to a more balanced content of cereal and legume grain in the final harvest, as confirmed by the significant trend of the Yield Ratio. However, in all mixed crop combinations included in the replacement design, LER_b_ and LER_p_ values still reflected a higher and lower performance than expected of barley and pea, respectively. Moreover, the ER index proved to be effective to identify traits that could be important to explain the differences in competitiveness observed between barley and pea.

Based on these results, the main target of growing barley and pea as mixed crop could be to maximize the yield of the legume exploiting advantages of intercropping such as reduced lodging of pea at harvest and better weed control than in pea pure crop, combined with a good performance of barley even at low sowing densities.

Furthermore, the 2017 field trial suggested that soil variability along the slope could have influenced the effectiveness of N fertilization on barley yield, and in 2018 the multivariate analysis of soil physicochemical parameters and asphyxial conditions differentiated the upslope and the downslope soils within the experimental field. The major effectiveness of the mineral N fertilization on barley in Soil2 (downslope), detected in 2018, was ascribed to the combination of urea fate and soil conditions. It is well-known that urea is initially transformed into NH_4_^+^ by hydrolysis promoted by urease and then into NO_3_^−^ by the nitrification process, and that NH_4_^+^ and NO_3_^−^, if not absorbed, have different fates depending on the soil moisture conditions and clay content^83–86^. In the profiles 4 and 5, the slight asphyxial conditions occurring at a shallow depth (30 cm) probably slowed down the nitrification process, favoring the persistence of NH_4_^+^ and consequently its adsorption on the abundant clay minerals (mostly vermiculite and hydroxy-Al interlayered vermiculite). Once adsorbed on clay minerals, NH_4_^+^ represents a NH_4_^+^ reservoir to be oxidized to NO_3_^−^ over a longer duration^87^, especially when the spring season moves forward and soil moisture decreases, so to allow O_2_ entering the soil. Because of this, the availability of urea-derived nitrates would have been higher in Soil2 than in the Soil1. This hypothesis is supported by (i) the absence of evidence suggesting soil erosion that could have translocated urea from upslope to downslope and (ii) the gentle slope that could not justify considerable subsurface water translocation of urea, also considering that urea is rapidly transformed into NH_4_^+^ and adsorbed on clay minerals. Therefore, a deeper evaluation of the relationships between soil and crops is proposed as an approach to be applied for more sustainable cropping systems. Thus, in this region where the soils are sub-alkaline with low organic matter content, hydrolysis of urea increases pH in proximity of the urea granule, with possible conversion of NH_4_^+^ to NH_3_^88^. As a consequence, urea application can lead to significant loss of N to the environment, particularly when it is not incorporated into the soil. Moreover, in accordance with recent studies^89^, because of the sub-alkaline soil pH values, available *P* content of the study area was generally low (mean available *P* = 10.1 mg kg^−1^). Therefore, increasing soil organic matter and decreasing soil pH can both increase the amount of available *P* and reduce N losses to the environment, contributing to crop improvement as well as to the sustainable management of the agro-ecosystem.

In 2018, the grain yield data analysis was firstly performed applying an ANOVA model for a split-plot design without including the Soil main effect and interactions and, as in 2017, the N fertilization and all interactions with Plant Teams and Mix were not significant. However, the pedological survey objectively justified the subdivision of the experimental field in upslope (Blocks 1 and 2) and downslope (Blocks 3 and 4) units. Then the ANOVA model including the Soil factor could have been applied resulting in significant effects of N fertilization on barley yield (as pure and in mixed crop) being detected only for Soil2.

Concerning with N fertilization, various plans were previously applied to compare the performance of barley–pea mixed crops with pure crops^41,46,58,68,90,91^.

Our results suggest that tailoring N levels based on the barley density in mixed crop combinations could reduce N supply as compared to cereal pure cropping, although a multiyear crop rotation plan must be defined, case by case, to effectively evaluate the overall effect of rotations based on mixed cropping on the use of N fertilizers use as compared to rotations based on pure crops.

Overall, growing pea in mixed cropping with barley was confirmed to be an effective strategy for more sustainable agricultural systems due to the better weed control and a reduced need of N fertilizers as compared to pure cropping systems. It is well known that growing pure grain legumes without using herbicides is quite difficult. This could be one of the main reasons why legumes have shown a progressive decrease in their diffusion as compared with cereals. Therefore, mixed cropping of pea with barley, or other less competitive cereals, at low density could be an effective strategy to expand the pea cultivation due to a better weed control than pea pure crops.

## Conclusions

Barley–pea intercropping combines two crops with very different behaviors in the mixed cropping system. The 50:50 barley:pea replacement design was not the best choice for the pedoclimatic conditions comprising fine-textured and sub-alkaline soils under the Mediterranean type of climate of central Italy. Differently, increasing the ratio between pea and barley densities provided a more balanced mixed cereal-legume grain ratio together with LER_tot_ ≥ 1 values.

However, the whole performance of the mixed crop in terms of grain yield, LER_tot_, and Yield Ratio would greatly increase if pea performance could be improved by widening the range of tested pea varieties, by selecting genotypes in specifically addressed pea plant breeding programs and by an optimization of the barley and pea sowing densities. These aspects deserve attention to extend pea cultivation in more sustainable agricultural systems, because of the higher effectiveness of barley–pea mixed cropping in controlling weeds than pea pure crops, if herbicides are not applied.

Finally, our results suggest that in hilly soils the evaluation of soil physicochemical properties and asphyxial conditions, coupled with differential N fertilization approaches, could be applied to optimize sustainable intensification programs of agricultural systems in the Mediterranean basin.

### Supplementary Information

Supplementary Tables.

Supplementary Information.

Supplementary Information.

Supplementary Information 1.

Supplementary Information 2.

## Data Availability

The datasets used and/or analysed during the current study available from the corresponding author on reasonable request.
