# Supplementary material for: Comparisons among barley–pea mixed crop combinations in a replacement design as related to N fertilization and soil variation
Source: Sci Rep. 2023 Sep 22;13:15825. doi: 10.1038/s41598-023-43050-9 (PMC10516871; doi:10.1038/s41598-023-43050-9)
Supplement: Supplementary file 1 — Supplementary Tables. [file 41598_2023_43050_MOESM1_ESM.pdf]

SUPPLEMENTARY TABLES

Manuscript Title: : Comparisons among barley-pea mixed crop combinations in a replacement design as related to N fertilization and soil variation.

Journal Name: Scientific Reports.

Authors: Stefano Tavoletti[a], Stefania Cocco[a], Giuseppe Corti[a,b]

[a] Stefano Tavoletti and Stefania Cocco: Dipartimento di Scienze Agrarie, Alimentari e Ambientali, Università Politecnica delle Marche, Ancona, Italy

[b] Giuseppe Corti: Dipartimento di Scienze Agrarie, Alimentari e Ambientali, Università Politecnica delle Marche, Ancona, Italy & Consiglio per la ricerca in agricoltura e l'analisi dell'economia agraria, Centro di ricerca Agricoltura e Ambiente, Rome, Italy.

Corresponding Author: Stefano Tavoletti, s.tavoletti@staff.univpm.it

Table S1. Setting of N fertilization supplied to each Plant Team at High and Low levels of N input in the 2018 trial. Only Mix1(50:50) was included in the 2017 trial.

**Plant Teams**	**N fertilization (kg N ha^{-1})**	
	**High N**	**Low N**
**Barley pure**	80.0	40.0
**Pea pure**	25.0	0.0
**Mix1 (barley 50%)**	40.0	20.0
**Mix2 (barley 33%)**	26.4	13.2
**Mix3 (barley 25%)**	20.0	10.0
**Mix4 (barley 20%)**	16.0	8.0

Table S2. Field trial 2017: ANOVA table (F test, mean square statistical significance: ** = *P* < 0.01, *** = *P* < 0.001).

Sources of Variation	df	Grain yield ($Mg\ ha^{-1}$)		Number of plants m^{-2}		Dry matter ($g\ m^{-2}$)		Spikes/pods $plant^{-1}$		Grain yield ($g\ plant^{-1}$)	
		Barley	Pea	Barley	Pea	Barley	Pea	Barley	Pea	Barley	Pea
Block (BL)	4	ns	ns	ns	ns	**	**	ns	ns	ns	ns
N Fert. (NF)	1	ns	ns	ns	ns	ns	ns	ns	ns	ns	ns
BL x NF (error 1)	4	**	ns	ns	ns	ns	ns	ns	ns	ns	**
Plant Team (PT)	5	***	***	***	***	***	***	***	***	***	***
PT x NF	5	ns	ns	ns	ns	ns	ns	ns	ns	ns	ns
Residual error	40										

Table S3. Field trial 2017: traits related to competition in mixed cropping. Multiple comparisons among plant team (PT) means (Tukey's HSD test), and comparison between observed and expected Equivalent Ratio (ER) values of barley and pea in each mixed crop combination (confidence interval, studentized range coefficient).

A) Number of plants per m^2

Plant Team	Barley[1]	ER_b[2]	Exp. ER_b	Pea[1]	ER_p[2]	Exp. ER_p
Pea1 (Hardy)				85.4 a		
Pea2 (Audit)				75.3 b		
Barley1 (Tea)	363 a					
Barley2 (Sunshine)	295 b					
Barley1-Pea1	160 c	0.45 ns	0.50	42.0 c	0.50 ns	0.50
Barley1-Pea2	166 c	0.47 ns	0.50	32.3 c	0.43 *	0.50
Barley2-Pea1	156 c	0.53 ns	0.50	37.4 c	0.44 ns	0.50
Barley2-Pea2	188 c	0.65 ***	0.50	36.3 c	0.49 ns	0.50

B) Dry matter yield (g m^{-2})

Plant Team	Barley[1]	ER_b[2]	Exp. ER_b	Pea[1]	ER_p[2]	Exp. ER_p
Pea1 (Hardy)				382.8 b		
Pea2 (Audit)				569.8 a		
Barley1 (Tea)	744.8 a					
Barley2 (Sunshine)	726.1 ab					
Barley1-Pea1	577.2 c	0.78***	0.50	76.2 c	0.29***	0.50
Barley1-Pea2	635.9 abc	0.87***	0.50	67.1 c	0.13***	0.50
Barley2-Pea1	642.2 abc	0.90***	0.50	63.8 c	0.22***	0.50
Barley2-Pea2	623.0 bc	0.86***	0.50	95.0 c	0.17***	0.50

C) Number of spikes/pods per plant

Plant Team	Barley[1]	ER_b[2]	Exp. ER_b	Pea[1]	ER_p[2]	Exp. ER_p
Pea1 (Hardy)				3.20 b		
Pea2 (Audit)				4.91 a		
Barley1 (Tea)	1.96 b					
Barley2 (Sunshine)	2.28 b					
Barley1-Pea1	3.15 a	1.62***	1.00	1.80 c	0.59***	1.00
Barley1-Pea2	3.04 a	1.56***	1.00	2.16 c	0.45***	1.00
Barley2-Pea1	3.27 a	1.47***	1.00	1.91 c	0.60***	1.00
Barley2-Pea2	2.83 a	1.26*	1.00	2.20 c	0.45***	1.00

D) Grain yield (g) per plant

Plant Team	Barley[1]	ER_b[2]	Exp. ER_b	Pea[1]	ER_p[2]	Exp. ER_p
Pea1 (Hardy)				4.87 a		
Pea2 (Audit)				5.40 a		
Barley1 (Tea)	2.05 b					
Barley2 (Sunshine)	2.19 b					
Barley1-Pea1	3.29 a	1.66***	1.00	2.40 b	0.51***	1.00
Barley1-Pea2	3.38 a	1.68***	1.00	2.14 b	0.40***	1.00
Barley2-Pea1	3.56 a	1.65***	1.00	1.87 b	0.40***	1.00
Barley2-Pea2	3.02 a	1.39**	1.00	2.32 b	0.43***	1.00

[1] Means followed by different letters are statistically different (Tukey's HSD test, $P \leq 0.05$)

[2] The difference between observed and expected (Exp.) LER values of barley and pea in each mix was tested by the confidence interval of each observed LER applying the studentized range coefficient: ns = not significant, *** = $P \leq 0.001$.

Table S4. Morphological description of the profiles of the barley-pea field trials. Agugliano Farm, Ancona (Italy).

Geology: thinly layered marine sediments – Landform: gentle slope (2-4%); Exposure: SSE – Altitude: from 48 to 46 m – Mean annual precipitation: 778 mm – Mean annual air temperature: 13.3°C; Warmest months: July and August (22.4°C) – Coldest month: January (4.5°C) – Drainage class: moderately drained. Vegetation cover: ≈5%, made of seedlings of herbaceous species. History of the field: it was probably cultivated with cereals until the early 1990s; then, it has been cultivated with cereals (mainly wheat); in 2017 there was soft wheat.
Soils: fine, mixed, mesic, Vertic Haplustepts (Soil Survey Staff, 2014).

Profile 1. Atop the slope. Slope: 4%. No artefacts.

	Depth, cm	Colour[a]	Structure[b]	Mottling[c]	Cutans[d]	Other observations
Ap1	0-9	2.5YR 4/4	3m abk&sbk, vfr	-	-	Skeleton: 5% (travertine and majolica)
Ap2	9-27	2.5YR 4/4	2m,co abk, fr	-		Skeleton: 5% (travertine and majolica)
Ap&Oi	27-31	2.5YR 3/3	1f,m abk&sbk, fr	-		Skeleton: 2-3% (travertine and majolica). The O material is wheat straw
Ap3	31-34	2.5YR 3/3	2m,co abk, fr	-		Skeleton: 2-3% (travertine and majolica)
Bw	34-67	2.5YR 4/4	2f,m abk&sbk, fr	-	3 faint	Skeleton: 5% (travertine and majolica). Fe-Mn nodules
BCg	67-95+	2.5YR 5/4	2m,co abk $\rightarrow$f pl, fr	++++, f-m, i	3 th	Skeleton: 5% (travertine and majolica). Fe-Mn nodules

Profile 2. About 20 m downslope. Profile 1. Slope: 3-4%. No artefacts.

	Depth, cm	Colour[a]	Structure[b]	Mottling[c]	Cutans[d]	Other observations
Ap1	0-7	2.5YR 4/3	3f,m abk&sbk, vfr			Skeleton: 5% (travertine and majolica)
Ap2	7-31	2.5YR 4/3	2-3f,m abk, fr			Skeleton: 5% (travertine and majolica)
Ap&Oi	31-35	2.5YR 3/3	1f,m abk&sbk, fr			Skeleton: 5% (travertine and majolica). The O material is wheat straw
Ap3	35-48	2.5YR 3/3	2m,co abk, fr		2 faint	Skeleton: 5% (travertine and majolica)
Bw	48-60	2.5YR 4/4	2f,m sbk&abk, fr	+, f	3 faint	Skeleton: 5% (travertine and majolica). Fe-Mn nodules
BCg	60-89+	2.5YR 5/4	2m,co abk $\rightarrow$f pl, fr	++++, f-m, i	3 th	Skeleton: 5% (travertine and majolica). Fe-Mn nodules

Profile 3. About 40 m downslope. Profile 2. Slope: 3%. No artefacts.

	Depth, cm	Colour[a]	Structure[b]	Mottling[c]	Cutans[d]	Other observations
Ap1	0-6	2.5YR 4/3	3f,m abk&sbk, fr			Skeleton: 5% (travertine and majolica)
Ap2	6-33	2.5YR 4/3	2m,co abk, fr			Skeleton: 5% (travertine and majolica)
Ap&Oi	33-36	2.5YR 3/2	1f,m sbk&abk, fr			Skeleton: 5% (travertine and majolica). The O material is wheat straw
Ap3	36-46	2.5YR 3/3	2m,co abk&sbk, fr	+, f		Skeleton: 5% (travertine and majolica)
Bw	46-56	2.5YR 4/4	2m,co abk, fr	++, f-m	3 faint	Skeleton: 5% (travertine and majolica). Fe-Mn nodules
BCg	56-92+	2.5YR 5/4	2m abk $\rightarrow$f pl, fr	++++, m, i	3 th	Skeleton: 5% (travertine and majolica). Fe-Mn nodules

Profile 4. About 60 m downslope. Profile 3. Slope: 2-3%. Presence of artefacts all along the profile.						
	Depth, cm	Colour[a]	Structure[b]	Mottling[c]	Cutans[d]	Other observations
Ap1	0-7	2.5YR 4/3	3f,m sbk&abk, vfr			Skeleton: 5% (travertine and majolica)
Ap2	7-30	2.5YR 4/3	2f,m abk, fr			Skeleton: 5% (travertine and majolica). Very few mottles
Ap&Oi	30-33	2.5YR 3/2	2f,m sbk&abk, fr	+, f	1 faint	Skeleton: 5% (travertine and majolica). The O material is wheat straw
Ap3	33-43	2.5YR 3/3	2m,co sbk&abk, fr	+, f	1-2 faint	Skeleton: 5% (travertine and majolica)
Bw	43-51	2.5YR 4/5	2co abk, fr	++, f-m	3 faint	Skeleton: 5% (travertine and majolica). Fe-Mn nodules
BCg	51-90+	2.5YR 5/4	2m abk →f pl, fr	++++, m-co, i	3 th	Skeleton: 5% (travertine and majolica). Fe-Mn nodules
Profile 5. About 80 m downslope. Profile 4. Slope: 2%. Presence of artefacts along the profile.						
	Depth, cm	Colour[a]	Structure[b]	Mottling[c]	Cutans[d]	Other observations
Ap1	0-6	2.5YR 4/3	3f,m sbk&abk, vfr			Skeleton: 5% (travertine and majolica)
Ap2	6-30	2.5YR 4/2	2f abk, fr			Skeleton: 5% (travertine and majolica)
Ap&Oi	30-33	2.5YR 3/3	2f,m sbk&abk, fr	+, f	1-2 faint	Skeleton: 5% (travertine and majolica). The O material is wheat straw
Ap3	33-41	2.5YR 3/3	2m,co sbk&abk, fr	++, f-m	2 faint	Skeleton: 5% (travertine & majolica).
Bw	41-50	2.5YR 4/5	2m,co abk, fr	++/+++, f-m	3 faint&th	Skeleton: 5% (travertine & majolica). Fe-Mn nodules
BCg	50-87+	2.5YR 5/4	2m abk →f pl, fr	++++, co, i	3 th	Skeleton: 5% (travertine & majolica). Fe-Mn nodules

[a] moist and crushed, according to the Munsell Soil Color Charts, 1954 edition.

[b] 1=weak, 2=moderate, 3=strong; f=fine, m=medium, c=coarse; sbk=subangular blocky, abk=angular blocky, pl=platy; fr=friable, vfr=very friable; →=breaking into.

[c] +=few (<2% of surface area), ++=common (2-20% of surface area), +++=many (20-40% of surface area), ++++=abundant (>40% of surface area); f=fine (<2 mm), m=medium (2-5 mm), co=coarse (5-20 mm); i=intricated.

[d] faint=<0.5 mm thick), thick=>0.5 mm thick; 1=very few (<5% of surface area), 2=few (5-25% of surface area), 3=common (25-50% of surface area).

Table S5. PCA results: loadings squared of physical (A) and chemical (B) variables. The sum of loading squared of variables measured for each horizon or for asphyxia depths, expressed as percentage of the PC eigenvalue (% Eigenvalue), is the percentage of total PC variance (eigenvalue) explained by each horizon and by asphyxia depths among physical variables. The % Eigenvalues explaining a higher amount of variance for each PC are highlighted in bold. The eigenvalue of each PC is reported as reference in the last row of the Table.

A) PHYSICAL VARIABLES

		PC1		PC2	
Horizon	Variables	Loading squared	% Eigenvalue	Loading squared	% Eigenvalue
Ap1	Clay%	0.468	12.9%	0.519	**22.1%**
	Silt%	0.212		0.095	
	Thickness	0.606		0.293	
Ap2	Clay%	0.413	13.4%	0.425	**30.1%**
	Silt%	0.532		0.421	
	Thickness	0.387		0.390	
Ap3	Clay%	0.601	11.1%	0.387	**24.6%**
	Silt%	0.136		0.414	
	Thickness	0.366		0.211	
Ap&Oi	Clay%	0.837	**23.9%**	0.140	11.6%
	Silt%	0.743		0.180	
	Thickness	0.797		0.156	
Bw	Clay%	0.699	**20.8%**	0.127	7.9%
	Silt%	0.591		0.000	
	Thickness	0.773		0.199	
Asphyxial	OXI	0.795	**17.8%**	0.144	3.7%
	UCTI	0.977		0.007	
PC Eigenvalue*		9.934		4.107	

B) CHEMICAL VARIABLES

		PC1		PC2	
Horizon	Variables	Loading squared	% Eigenvalue	Loading squared	% Eigenvalue
Ap1	SOC	0.779	**30.5%**	0.014	1.5%
	NH4-N	0.647		0.005	
	Avail. P	0.791		0.099	
	pH	0.385		0.001	
Ap2	SOC	0.473	13.8%	0.501	**28.0%**
	NH4-N	0.165		0.677	
	Avail. P	0.509		0.366	
	pH	0.029		0.648	
Ap3	SOC	0.842	**31.9%**	0.120	11.4%
	NH4-N	0.717		0.196	
	Avail. P	0.597		0.218	
	pH	0.568		0.357	
Ap&Oi	SOC	0.257	13.6%	0.547	**24.8%**
	NH4-N	0.644		0.300	
	Avail. P	0.242		0.452	
	pH	0.018		0.642	
Bw	SOC	0.578	10.3%	0.273	**34.3%**
	NH4-N	0.163		0.793	
	Avail. P	0.107		0.847	
	pH	0.030		0.768	
PC Eigenvalue*		8.543		7.825	

*PC Eigenvalue = Sum of Loading squared.

Table S6. Field trial 2018. ANOVA results (ANOVA Model A) for traits analysed on: A) pure crops (grain yield, number of plants per m^2, dry matter (DM) yield per m^2, number of spikes or pods per plant, grain yield per plant), and B) pure and mixed crops (dry matter of weeds).

Sources of variation	A) Pure crops[2]						B) Pure & Mixed crops[2]	
	df	Grain yield	Plants m^{-2}	DM g m^{-2}	Spikes/Pods $plant^{-1}$	Yield $Plant^{-1}$	df	Weeds DM g m^{-2}
Soil	1	ns	ns	**	*	ns	1	ns
Blocks [Soil] error1	2	ns	ns	ns	ns	ns	2	ns
Nitrogen Fert. (NF)	1	ns	ns	*	ns	ns	1	ns
NF x Soil	1	*	ns	*	ns	ns	1	ns
Blocks x NF [Soil] error2	2	ns	ns	ns	ns	ns	2	ns
Plant Teams (PT)[1]	2	***	***	***	***	***	10	***
PT x Soil	2	*	ns	ns	ns	ns	10	ns
PT x NF	2	**	ns	*	ns	ns	10	*
PT x Soil x NF	2	*	ns	ns	ns	ns	10	ns
Residual error	8							

[1] Levels of Plant Teams: A) 3 pure crops (Barley1, Pea1, Pea3), and B) 11 (3 pure + 8 mixed crops).
[2] Statistical significances (F test): ns = not significant, * = $P < 0.05$, ** = $P < 0.01$, *** = $P < 0.001$.

Table S7. Field trial 2018. Evaluation of pure crop (PT) as main factor and of PT x Soil x NF interaction for (A) grain yield (Mg ha^{-1}) and (B-E) traits collected on sampling areas.

	**PT**	**PT x Soil x NF interaction**					
**A) Grain yield (Mg ha^{-1})**		Soil1			Soil2		
Pure crops	Yield	Low N	High N	Sign.	Low N	High N	Sign.
Pea3 (Astronaute)	3.15 a	3.31	3.15	ns	3.04	3.11	ns
Pea1 (Hardy)	2.83 b	2.86	2.63	ns	2.86	2.97	ns
Barley1 (Tea)	2.57 c	2.34	2.45	ns	2.21	3.27	***

**B) Number of Plants m^{-2}**	# of	Soil1			Soil2		
Pure crops	plants	Low N	High N	Sign.	Low N	High N	Sign.
Pea3 (Astronaute)	75.6 b	76.7	76.7	ns	65.4	83.8	ns
Pea1 (Hardy)	85.4 b	88.4	89.6	ns	83.8	80.0	ns
Barley1 (Tea)	434.7 a	457.9	418.3	ns	416.7	445.8	ns

**C) Dry Matter (DM. g m^{-2})**		Soil1			Soil2		
Pure crops	DM	Low N	High N	Sign.	Low N	High N	Sign.
Pea3 (Astronaute)	225.6 b	192.8	235.4	ns	165.9	308.4	***
Pea1 (Hardy)	209.6 b	220.7	199.0	ns	206.1	212.5	ns
Barley1 (Tea)	336.2 a	306.5	335.4	ns	302.9	399.9	**

**D) Number of spikes or pods per plant**	# of spikes/	Soil1			Soil2		
Pure crops	pods	Low N	High N	Sign.	Low N	High N	Sign.
Pea3 (Astronaute)	-----	6.26	6.06	ns	5.64	6.64	ns
Pea1 (Hardy)	-----	4.89	4.60	ns	4.94	5.88	ns
Barley1 (Tea)	-----	1.44	1.43	ns	1.38	1.65	ns

**E) Grain Yield (g) per Plant**		Soil1			Soil2		
Pure crops	Yield	Low N	High N	Sign.	Low N	High N	Sign.
Pea3 (Astronaute)	-----	5.28	4.76	ns	5.51	4.82	ns
Pea1 (Hardy)	-----	4.40	4.32	ns	4.42	5.06	ns
Barley1 (Tea)	-----	0.60	0.58	ns	0.51	0.79	ns

Table S8. Field trial 2018. ANOVA results (ANOVA Model B) for traits analysed on mixed crops (Grain yield. number of plants per m^2. dry matter yield per m^2. number of spikes or pods per plant, grain yield per plant).

Sources of variation	df	Grain yield			Number of plants m^{-2}		Dry matter ($g\ m^{-2}$)		Spikes/Pods $plant^{-1}$		Grain yield $plant^{-1}$	
		Barley	Pea	Total	Barley	Pea	Barley	Pea	Barley	Pea	Barley	Pea
Soil	1	*	ns	ns	ns	*	ns	ns	**	ns	ns	ns
Blocks [Soil]	2	ns	ns	ns	ns	ns	ns	ns	ns	ns	ns	ns
Nitrogen Fert. (NF)	1	***	ns	ns	ns	*	ns	ns	*	ns	ns	ns
NF x Soil	1	**	ns	ns	ns	ns	ns	ns	ns	ns	ns	ns
Blocks x NF [Soil]	2	ns	ns	ns	ns	ns	ns	ns	ns	ns	ns	ns
Mix[1]	3	***	***	ns	***	***	***	***	***	ns	***	**
Plant Teams (PT)[2]	1	***	ns	**	ns	**	ns	ns	**	***	***	***
Mix x PT	3	ns	ns	ns	ns	ns	ns	ns	ns	ns	ns	ns
Mix x Soil	3	***	ns	ns	ns	ns	ns	ns	ns	ns	ns	ns
Mix x NF	3	***	ns	ns	ns	ns	ns	ns	ns	ns	ns	*
Mix x Soil x NF	3	**	ns	ns	ns	ns	ns	ns	ns	ns	ns	ns
PT x Soil	1	*	ns	ns	ns	ns	ns	ns	ns	ns	ns	ns
PT x NF	1	ns	ns	ns	ns	ns	ns	ns	ns	ns	ns	ns
PT x Soil x NF	1	ns	ns	ns	ns	ns	ns	ns	ns	ns	ns	ns
Mix x PT x Soil	3	ns	ns	ns	ns	ns	ns	ns	*	ns	ns	ns
Mix x PT x NF	3	ns	ns	ns	ns	ns	ns	ns	ns	ns	ns	**
Mix x PT x Soil x NF	3	***	ns	ns	ns	ns	ns	ns	ns	ns	ns	ns
Residual error	28											

[1] Mixed crop combinations: Mix1(50:50). Mix2(33:67). Mix3(25:75). Mix4(20:80).
[2] Plant Teams: Barley1- Pea1. Barley1-Pea3
Statistical significance (F test): ns = not significant. * = $P < 0.05$. ** = $P < 0.01$. *** = $P < 0.001$.

Table S9. Field trial 2018. Multiple comparisons (Tukey's HSD test. $\alpha = 0.05$) among average Yield Ratios (Barley Yield / Pea Yield) of mixed crops in the two Soils at High and Low N levels. Yield Ratio means are reported in the original scale. after exponential inverse transformation of natural Log values.

**Yield ratio**		**Mix1**	**Mix2**	**Mix3**	**Mix4**
**Soil2**	**High N**	3.40 a	1.64 cde	1.10 efg	1.00 g
	**Low N**	1.98 cd	1.42 defg	0.99 g	0.91 g
**Soil1**	**High N**	2.60 b	1.60 cdef	1.05 fg	0.92 g
	**Low N**	2.18 bc	1.44 defg	0.97 g	0.87 g

Table S10. Field trial 2018: mixed crops. Traits collected on sampling areas and related to crop competition in mixed cropping: multiple comparisons among means (Tukey's HSD test) and comparison between observed and expected Equivalent Ratio (ER) values.

A) Number of plants m^{-2}

Mix	Barley[1]	ER_b[2]	Exp ER_b	Pea[1]	ER_p[2]	Exp ER_p
Mix1	217.6 a	0.51ns	0.50	38.4 c	0.48 ns	0.50
Mix2	155.2 b	0.36ns	0.33	55.6 b	0.69 ns	0.67
Mix3	101.2 c	0.24ns	0.25	62.1 ab	0.77 ns	0.75
Mix4	100.1 c	0.24ns	0.20	66.5 a	0.82 ns	0.80

B) Dry matter yield (g m^{-2})

Mix	Barley[1]	ER_b[2]	Exp ER_b	Pea[1]	ER_p[2]	Exp ER_p
Mix1	295.4 a	0.88***	0.50	67.4 b	0.32***	0.50
Mix2	258.2 ab	0.78***	0.33	97.4 a	0.46***	0.67
Mix3	214.7 bc	0.65***	0.25	117.7 a	0.57***	0.75
Mix4	194.5 c	0.59***	0.20	120.8 a	0.58***	0.80

C) Number of spikes (barley) or pods (pea) per plant

Mix	Barley[1]	ER_b[2]	Exp ER_b	Pea[1]	ER_p[2]	Exp ER_b
Mix1	1.78 c	1.21***	1	3.80 a	0.68***	1
Mix2	2.14 b	1.46***	1	3.62 a	0.65***	1
Mix3	2.54 a	1.73***	1	3.92 a	0.70***	1
Mix4	2.33 ab	1.60***	1	3.87 a	0.69***	1

D) Grain yield (g) per plant

Mix	Barley[1]	ER_b[2]	Exp ER_b	Pea[1]	ER_p[2]	Exp ER_p
Mix1	1.11 c	1.81***	1	3.07 ab	0.64***	1
Mix2	1.32 b	2.15***	1	2.76 b	0.58***	1
Mix3	1.53 a	2.51***	1	3.40 a	0.71***	1
Mix4	1.59 a	2.60***	1	3.20 a	0.66***	1

[1] Means followed by different letters are statistically different (Tukey's HSD test. $P < 0.05$)

[2] The difference between observed and expected (Exp.) ER values of barley and pea in each mix was tested by the confidence interval of observed ER applying the studentized range coefficient: ns = not significant. *** = $P < 0.001$.
