# Supplementary material for: Comparisons among barley–pea mixed crop combinations in a replacement design as related to N fertilization and soil variation
Source: Sci Rep. 2023 Sep 22;13:15825. doi: 10.1038/s41598-023-43050-9 (PMC10516871; doi:10.1038/s41598-023-43050-9)
Supplement: Supplementary file 2 — Supplementary Information. [file 41598_2023_43050_MOESM2_ESM.pdf]

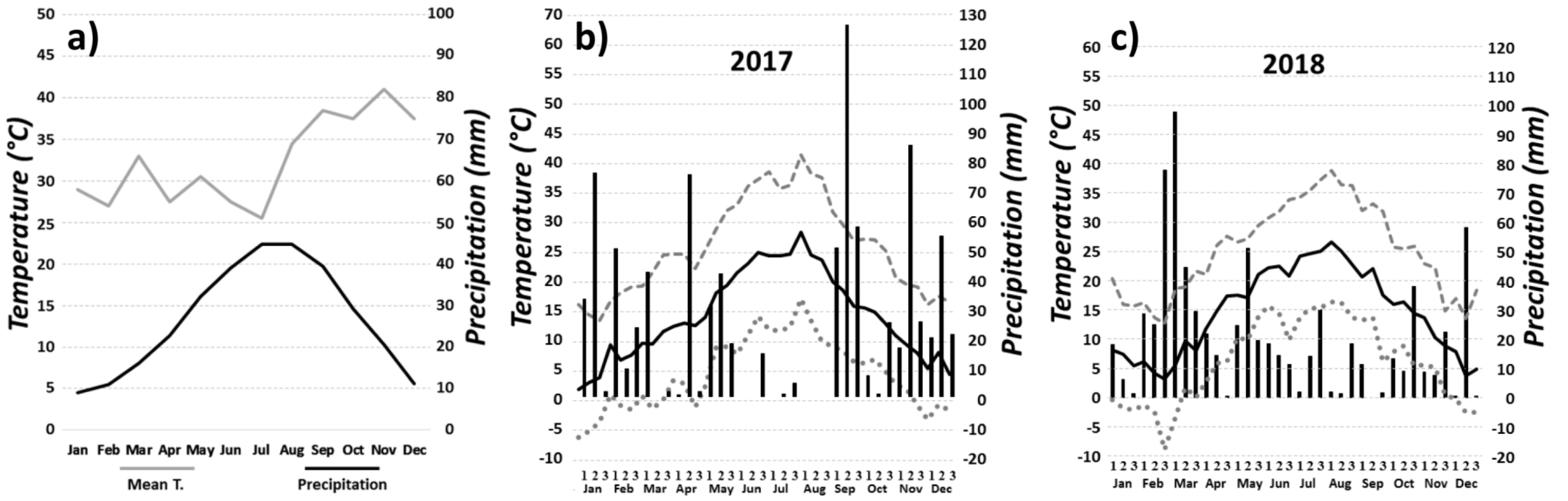

Figure S1. Climatic diagram for UNIVPM Experimental Farm (Agugliano, Ancona, Italy) plotted for the thirty-year period 1990-2019 (a); total precipitation (bars, mm), mean air temperature (continuous line, °C), and absolute minimum (dotted line) and maximum (dashed line) temperature (°C) for ten-day periods for the years 2017 (b) and 2018 (c).
