# Supplementary material for: Comparisons among barley–pea mixed crop combinations in a replacement design as related to N fertilization and soil variation
Source: Sci Rep. 2023 Sep 22;13:15825. doi: 10.1038/s41598-023-43050-9 (PMC10516871; doi:10.1038/s41598-023-43050-9)
Supplement: Supplementary file 3 — Supplementary Information. [file 41598_2023_43050_MOESM3_ESM.pdf]

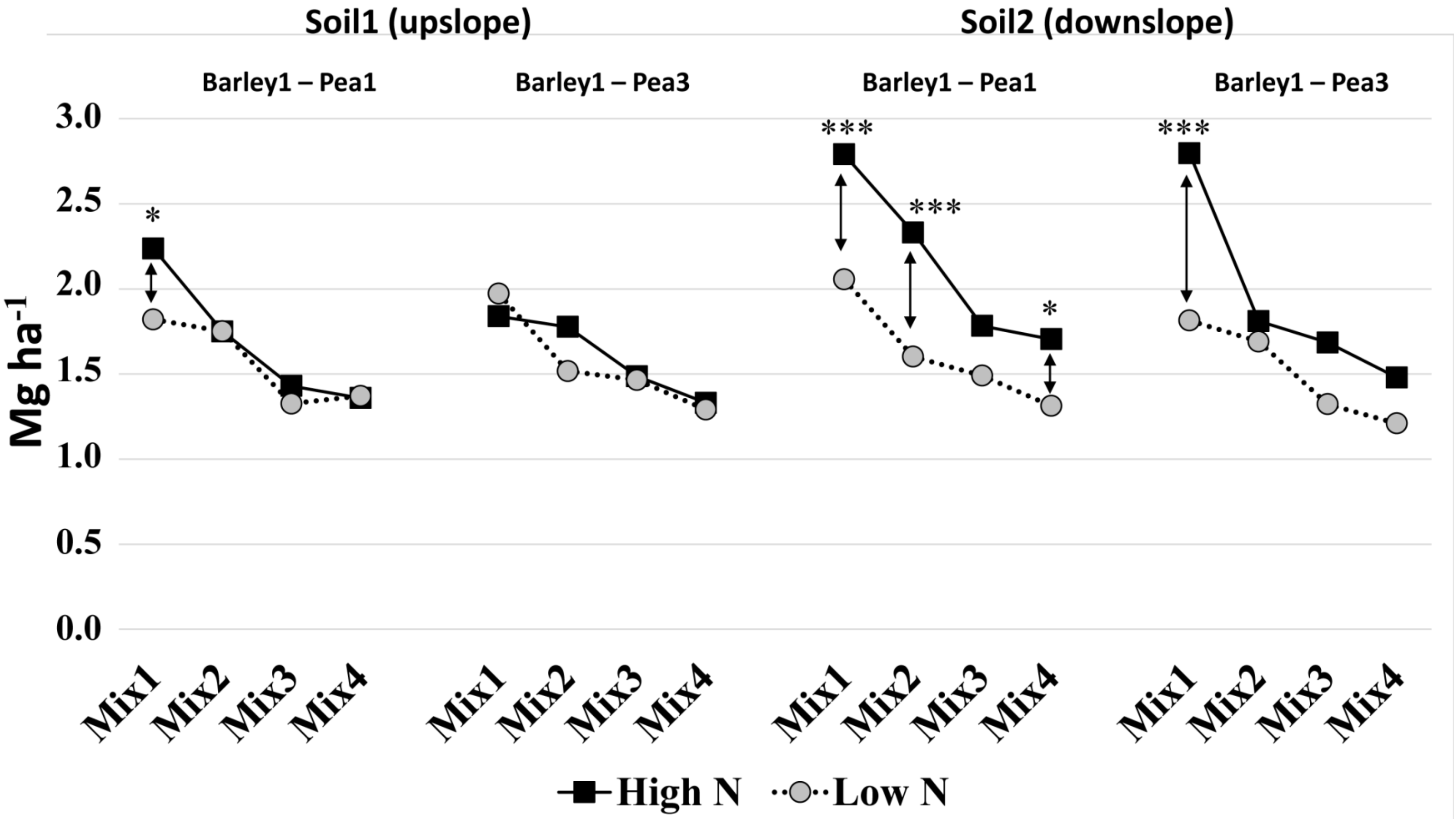

Figure S2. Barley yield (Mg ha^{-1}) in all mixed crop combinations evaluated in Soil1 and Soil2 at High and Low N levels (SOIL x NF x MIX x PT interaction). The LER$_b$ values of each mixed crop are shown close to the respective barley mean yield. For each barley-pea mix, significant differences (Contrasts, Bonferroni correction) between High and Low N levels, within Soil1 and Soil2, are highlighted (* = $P < 0.05$, *** = $P < 0.001$).
