# Supplementary material for: Comparisons among barley–pea mixed crop combinations in a replacement design as related to N fertilization and soil variation
Source: Sci Rep. 2023 Sep 22;13:15825. doi: 10.1038/s41598-023-43050-9 (PMC10516871; doi:10.1038/s41598-023-43050-9)
Supplement: Supplementary file 4 — Supplementary Information 1. [file 41598_2023_43050_MOESM4_ESM.pdf]

Supplementary text file S1

Manuscript Title: : Comparisons among barley-pea mixed crop combinations in a replacement design as related to N fertilization and soil variation.

Journal Name: Scientific Reports.

Authors: Stefano Tavoletti[a], Stefania Cocco[a], Giuseppe Corti[a,b]

[a] Stefano Tavoletti and Stefania Cocco: Dipartimento di Scienze Agrarie, Alimentari e Ambientali, Università Politecnica delle Marche, Ancona, Italy

[b] Giuseppe Corti: Dipartimento di Scienze Agrarie, Alimentari e Ambientali, Università Politecnica delle Marche, Ancona, Italy & Consiglio per la ricerca in agricoltura e l'analisi dell'economia agraria, Centro di ricerca Agricoltura e Ambiente, Rome, Italy.

Corresponding Author: Stefano Tavoletti, s.tavoletti@staff.univpm.it

ANOVA Model A.

Field trial 2018: ANOVA model applied to analyze **pure crop** yields and traits measured on the sampling area within each plot (number of plants per m^2, crop dry matter yield per m^2, number of spikes per plant, grain yield per plant for barley, number of plants per m^2 and crop dry matter yield per m^2 for pea).

The same model was applied to analyze weeds dry matter (g m^{-2}) including both pure and mixed crops.

$$y_{ijkl} = \mu + \tau_i + \beta(\tau)_{ji} + \alpha_k + \alpha\tau_{ki} + \varepsilon_{ijk} + \gamma_l + \tau\gamma_{il} + \alpha\gamma_{kl} + \tau\alpha\gamma_{ikl} + \varepsilon_{ijkl}$$

where,

y_{ijkl} = measured traits: pure crop grain yield, number of plants per m^2, crop dry matter yield per m^2, number of spikes per plant, grain yield per plant, weeds dry matter (g m^{-2})

μ= overall mean

τ_i = Soil (i = 1,2)

$\beta(\tau)_{ji}$ = error 1, Blocks nested within Soil (j=1,2)

α_k = Nitrogen Fertilization (NF) effect, (k = 1,2)

$\alpha\tau_{ki}$ = NF x Soil interaction

ε_{ijk} = error2 (Blocks x NF, nested within Soil)

γ_l = Plant Team (PT), where

  l = 1,⋯,3 (1 barley + 2 pea varieties) for pure crop grain yield, number of plants per m^2, crop dry matter yield per m^2, number of spikes per plant, and grain yield per plant,

  l = 1,⋯,11 (3 pea pure + 8 mixed crop combinations) for weeds dry matter (g m^{-2})

$\tau\gamma_{il}$ = Soil x PT interaction

$\alpha\gamma_{kl}$ = NF x PT interaction

$\tau\alpha\gamma_{ikl}$ = Soil x NF x PT interaction

ε_{ijkl} = residual error

ANOVA Model B. ANOVA model applied to analyze barley, pea, and total yield of **mixed crops** in the 2018 field trial.

$$y_{ijkl} = \mu + \tau_i + \beta(\tau)_{ji} + \alpha_k + \tau\alpha_{ik} + \varepsilon_{ijk} + \gamma_l + \varpi_m + \gamma\varpi_{lm} + \tau\gamma_{il} + \alpha\gamma_{kl} + \tau\alpha\gamma_{ikl} + \tau\varpi_{jm} + \alpha\varpi_{km} + \tau\alpha\varpi_{ikm} + \tau\gamma\varpi_{ikm} + \alpha\gamma\varpi_{klm} + \tau\alpha\gamma\varpi_{iklm} + \varepsilon_{ijklm}$$

where,

y_{ijkl} = measured traits: grain yield of each species, total grain yield, number of plants per m^2, crop dry matter yield per m^2, number of spikes per plant, grain yield per plant, LER_b, LER_p LER_{tot} and ER.

μ= overall mean

τ_i = Soil effect (i = 1,2)

$\beta(\tau)_{ji}$ = error 1, Blocks nested within Soil (j=1,2)

α_k = Nitrogen Fertilization (NF) effect (k = 1,2)

$\alpha\tau_{ki}$ = NF x Soil interaction

ε_{ijk} = error2 (Blocks x NF nested within S)

γ_l = Plant Team (PT) (l = 1,2: Barley1-Pea1, Barley1-Pea3)

ϖ_m = MIX (m = 1,…,4: Mix1, Mix2, Mix3, Mix4)

$\gamma\varpi_{lm}$ = PT x MIX interaction

$\tau\gamma_{il}$ = Soil x PT interaction

$\alpha\gamma_{kl}$ = NF x PT interaction

$\tau\alpha\gamma_{ikl}$ = Soil x NF x PT interaction

$\tau\varpi_{jm}$ = Soil x MIX interaction

$\alpha\varpi_{km}$ = NF x MIX interaction

$\tau\alpha\varpi_{ikm}$ = Soil x NF x MIX interaction

$\tau\gamma\varpi_{ilm}$ = Soil x PT x MIX interaction

$\alpha\gamma\varpi_{klm}$ = NF x PT x MIX interaction

$\tau\alpha\gamma\varpi_{iklm}$ = Soil x NF x PT x MIX interaction

ε_{ijkl} = residual error
