# Supplementary material for: Comparisons among barley–pea mixed crop combinations in a replacement design as related to N fertilization and soil variation
Source: Sci Rep. 2023 Sep 22;13:15825. doi: 10.1038/s41598-023-43050-9 (PMC10516871; doi:10.1038/s41598-023-43050-9)

Supplementary text file S2

Manuscript Title: : Comparisons among barley-pea mixed crop combinations in a replacement design as related to N fertilization and soil variation.

Journal Name: PLANT and SOIL.

Authors: Stefano Tavoletti[a], Stefania Cocco[a], Giuseppe Corti[a,b]

[a] Stefano Tavoletti and Stefania Cocco: Dipartimento di Scienze Agrarie, Alimentari e Ambientali, Università Politecnica delle Marche, Ancona, Italy

[b] Giuseppe Corti: Dipartimento di Scienze Agrarie, Alimentari e Ambientali, Università Politecnica delle Marche, Ancona, Italy & Consiglio per la ricerca in agricoltura e l'analisi dell'economia agraria, Centro di ricerca Agricoltura e Ambiente, Rome, Italy.

Corresponding Author: Stefano Tavoletti, s.tavoletti@staff.univpm.it

ANOVA table for model WITHOUT the Soil factor applied to barley grain yield in mixed cropping.

Sources of variation	df	Sign.	P value
Blocks	3	ns	P = 0.4906
N Fertilization (NF)	1	ns	P = 0.0891
**Blocks x NF (error1)**	**3**	*******	**P = 0.0002**
Mix	3	***	P < 0.0001
Plant Team (PT)	1	*	P = 0.0152
Mix x PT	3	ns	P = 0.6422
Mix x NF	3	*	P = 0.0245
PT x NF	1	ns	P = 0.2610
Mix x NF x PLT	3	ns	P = 0.8466
Residual error	42		

The Blocks main factor was not significant, as well as the NF main factor, whereas the BL x NF (error1) was highly significant (*P*=0.0002). This result suggested, for barley yield, a different average response among blocks to N fertilization, that is well represented by the BL x NF figure shown below (multiple comparisons among barley mean grain yield recorded for each Block at High N and Low N levels).

It shows clear Blocks x NF interaction. In particular, NF was effective in increasing mean barley yield only in Blocks 3 and 4 located downslope, whereas no significant difference was observed in Blocks 1 and 2 located upslope. This graph clearly shows how barley grain yield reflected the PCA and CA results on soil physicochemical properties. Therefore, the soil investigation justified that the Soil factor and all its interactions could have been included in the ANOVA model applied to 2018 results.

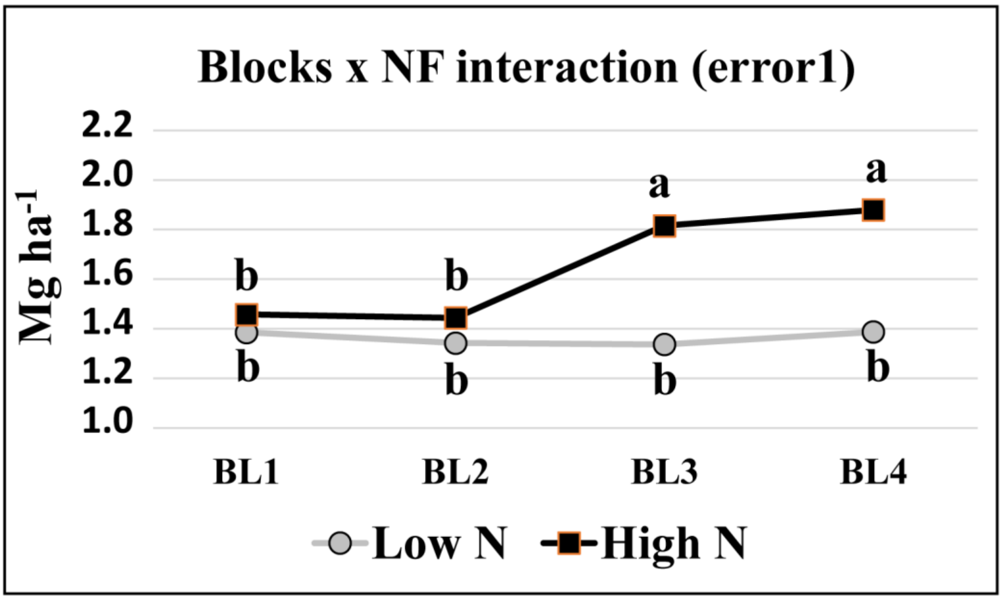

Supplement: Supplementary file 5 — Supplementary Information 2. [file 41598_2023_43050_MOESM5_ESM.pdf]
